# Pictorial Imaging-Histopathology Correlation in a Rabbit with Hepatic VX2 Tumor Treated by Transarterial Vascular Disrupting Agent Administration

**DOI:** 10.7150/ijms.46165

**Published:** 2020-08-25

**Authors:** Jintong He, Chong Liu, Tian Li, Yewei Liu, Shuncong Wang, Jian Zhang, Lei Chen, Chao Wang, Yuanbo Feng, Giuseppe Floris, Zhiqiang Wang, Xian Zhang, Liwen Zhao, Yue Li, Haibo Shao, Yicheng Ni

**Affiliations:** 1 Department of Interventional Radiology, the First Hospital of China Medical University, Shenyang 110001, China; 2 Shanghai Key Laboratory of Molecular Imaging, Shanghai University of Medicine and Health Sciences, Shanghai 201318, China; 3 KU Leuven, Biomedical Group, Campus Gasthuisberg, 3000 Leuven, Belgium; 4 Laboratory of Translational Medicine, Jiangsu Province Academy of Traditional Chinese Medicine, Nanjing 210028, China; 5 Nanjing Sanhome Pharmaceutical Co. Ltd., Nanjing 211135, China

**Keywords:** Cancer treatment, vascular disrupting agents (VDAs), C118P, Rabbit, VX2 carcinoma

## Abstract

Cancer vasculature is immature, disorganized and hyperpermeable and can serve as a target for anti-cancer therapies. Vascular disrupting agents (VDAs) are tubulin protein binding and depolymerizing agents that induce rapid tumoral vascular shutdown and subsequent cancer necrosis. However, two clinical problems exist with all VDAs, i.e. 1) incomplete anticancer effect and 2) dose-dependent toxicity.

To tackle these problems, in our ongoing research, a novel VDA C118P is applied by transarterial administration of half the intravenous dose in rabbits with implanted VX2 liver tumor to assess its therapeutic efficacy.

Nearly complete tumor necrosis was achieved by only a single arterial dose of C118P at 5 mg/kg, which was documented in a representative case by in vivo digital subtraction arteriogram (DSA) and magnetic resonance imaging (MRI), and further confirmed by ex vivo microangiogram and histopathology.

This convincing and promising preliminary outcome would warrant further comprehensive studies to explore the potentials of VDAs by transarterial administration either in mono-drug or in combination for management of solid cancers.

## Introduction

Vasculature of cancers is known to be immature, disorganized and hyperpermeable, which has become a target for developing anti-cancer therapies. Vascular disrupting agents (VDAs) such as the most investigated combretastatin A-4-phosphate (CA4P; fosbretabulin) are a class of tubulin protein binding and depolymerizing agents, which induce rapid tumoral vascular shutdown and subsequent cancer cell death among virtually all solid malignancies 1,2. CA4P has been undergone phase I-III clinical trials in both single and combinatory settings for a variety of cancer types 3-6. However, these clinical trials failed to achieve satisfactory outcomes that otherwise would allow the FDA approval for entering cancer market 7.

A key problem with VDAs is being never capable of complete tumor necrosis with remnant viable cancer cells that inevitably result in cancer regrowth 1-7. To tackle this bottleneck problem, a highly synergetic dual-targeting broad-spectrum theragnostic strategy called OncoCiDia has been developed 7-10 and is now under early veterinary and human clinical trials 11-13. In OncoCiDia, on top of a single intravenous (iv) dosage of VDA, a single iv dose of radioiodinated necrosis avid agent such as hypericin (^131^I-Hyp) is followed the next day to facilitate targeted persistent radionuclide therapy to sterilize remnant oncocytes, and simultaneous scintigraphy imaging on the primary and/or metastatic solid cancers 7-10.

Despite favorable safety and efficacy with systemic OncoCiDia, currently we are elaborating locoregional OncoCiDia through interventional transcatheter arterial drug deliveries for certain indications, thus to substantially reduce the amount of the two applied agents but meanwhile to make OncoCiDia even safer and more efficacious. To our knowledge, intra-arterially delivered VDA was reported only in combinatory manner for experimental therapy of liver malignancies 14. The anticancer competence of a VDA given by arterial catheter for liver cancers has never been evaluated or reported, most likely due to the influence from the previously documented unfavorable result in rats with renal tumor allograft 15. Indeed, repeated iv doses of VDAs are known to cause cardiovascular toxicity and other reversible side effects 16, but imaging guided transarterial interventions have been practiced for decades as clinical routines, which can be utilized for a more targeted cancer drug delivery.

Therefore, as a first step to realize OncoCiDia via an arterial access with a reduced VDA dose, i.e. locoregional OncoCiDia, we are now conducting an animal experiment in rabbits with intrahepatically implanted VX2 carcinoma by transcatheter arterial infusion of a novel VDA called C118P 17 in order to evaluate both the safety and efficacy of this agent at a smaller arterial dosage.

A single case report article is not uncommon in the literature especially when something unprecedentedly presents 18. Similarly, while working in progress, we would like already to showcase a typical example with complete imaging-histopathology correlative evidences to demonstrate that only once intra-arterially administered VDA can efficiently necrotize the solid liver cancer in rabbits, which, together with the upcoming more comprehensive preclinical data, may pave the path for future development and applications of locoregional OncoCiDia.

## Materials and Methods

### Applied VDA

**Figure 1:**
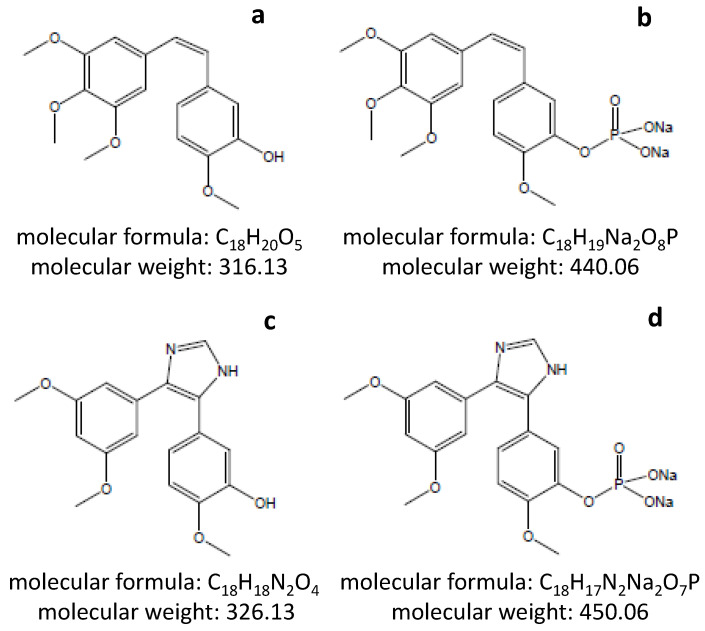
Chemical structures of CA4 (a), CA4P (b), C118 (c) and C118P (d) as well as their molecular formulae and masses

Analogous to CA4 (Fig 1A), the most potent naturally occurring combretastatins, and its hydrophilic prodrug CA4P (Fig 1B), the modified molecules of C118 (Fig 1C) and its phosphate prodrug C118P (Figure 1D) are all shown in Figure 1 to display their chemical properties. The hydrophilic prodrug C118P applied in this study was developed and supplied by Sanhome Pharmaceutical Co. Ltd. (Nanjing, China), which is currently under Phase Ib clinical trial for the safety and efficacy in treatment of patients with solid tumors 17. The maximal tolerated dose (MTD) has not been reached yet but should be > 113 mg/m^2^ (Wang C. personal communication) in contrast to MTD of CA4P at 68 mg/m^2^ 1-6. This human dose for repeated daily iv infusion can be converted to ~10 mg/kg in rabbits using a recommended equation 19. Considering future novel approach of locoregional OncoCiDia with a single dose of intrahepatic arterial infusion, we applied a reduced dose of 5 mg/kg in this study to minimize possible side effects.

### Tumor Model

**Figure 2:**
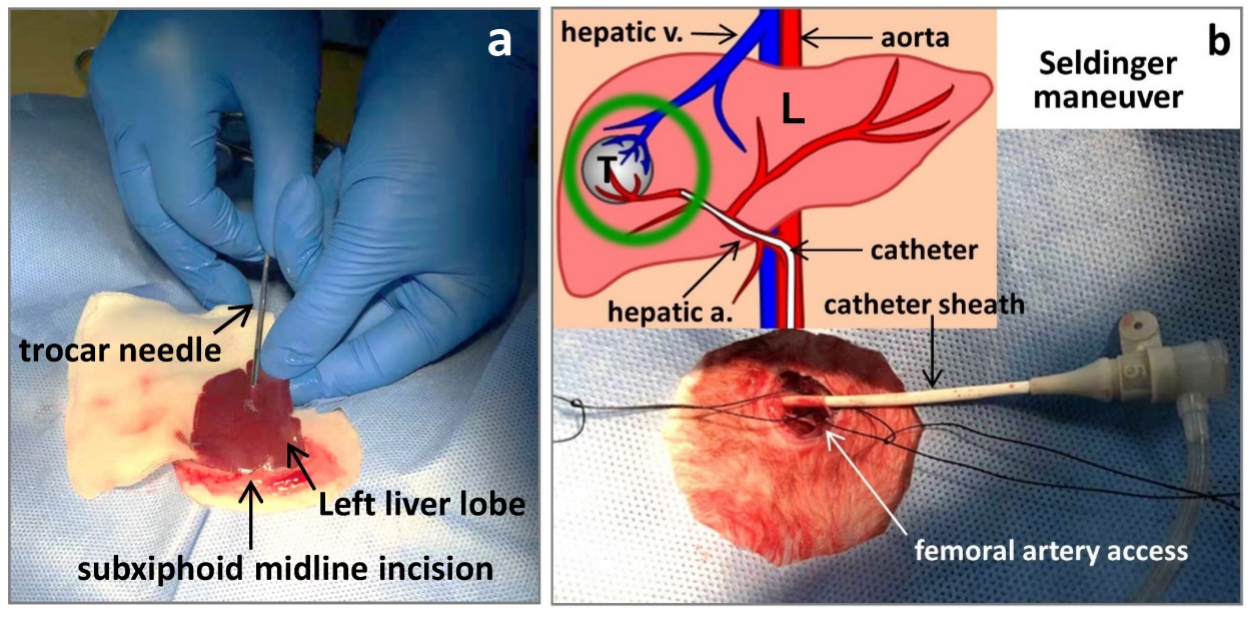
Making rabbit VX2 liver tumor model by laparotomy (a); and transcatheter hepatic arterial infusion of C118P by Seldinger technique (b) where L,T, a. and v. stand for the liver, tumor, artery and vein, respectively.

This animal study is in compliance with national regulations for animal welfare and approved by the Institutional Ethical Committee for Animal Care and Use. New Zealand white rabbits weighing 3.5kg were employed in this study and they were anesthetized with iv injection of a 3% pentobarbital sodium solution (Sigma) at a dose of 10 mg/kg via marginal aural vein for imaging, surgical and interventional procedures. One rabbit with subcutaneous VX2 tumors growing at its hind limb served as a donor. Under aseptic conditions, one tumor was resected and the incision was sutured. The tumor was cleaned by phosphate buffered solution (PBS) and chopped into 1.0 mm^3^ minute cubes. For making liver tumor models, after shaving hair and sterilizing skin, the rabbit was placed in supine position and given a small subxiphoid midline incision. The left lateral liver lobe was gently externalized onto a piece of PBS soaked gauze, ready for tumor implantation. A freshly harvested VX2 tumor tissue cube was loaded in the tip of a 16G trocar needle, which was punctured through liver capsule into the parenchyma about 1cm in depth. The tumor tissue was engrafted by pushing the needle core (Fig 2a) and after withdrawing the needle, a drop of tissue glue (Histoacryl^®^, Braun, Germany) was applied at puncture site to stop bleeding. The liver lobe was re-positioned into abdominal cavity, followed by layered suture to close the incision. Penicillin of 200,000U was injected intramuscularly per day for 3 days to prevent infection. This rabbit model of solitary liver tumor was virtually 100% successful.

### MR imaging

Magnetic resonance imaging (MRI) was performed at a 3.0T MRI unit (Signa HDx, General Electric Medical Systems, USA) with a maximum gradient strength of 40 mT/m equipped with a HD Quadrature Knee/Foot Coil 10. To keep a symmetrical supine position, the rabbit was fixed in a plastic holder which was matched with the knee coil in diameter. Coronal, sagittal, and axial pilot images were first obtained to localize subsequent MRI acquisitions. For each imaging sequence, 18 axial images were obtained with a section thickness of 3.0 mm and an intersection gap of 1.0 mm. Fast spin-echo (FSE) T2 weighted imaging (T2WI) and spin-echo (SE) T1 weighted imaging (T1WI) were performed first; and then contrast enhanced T1 weighed images (CE-T1WI) were acquired after intravenous bolus injection of Dotarem® (Gd-DOTA, Guerbet, France) at 0.2 mmol/kg. Two weeks after implantation, rabbits were screened for tumor growth by T2WI and CE-T1WI. When the liver tumor diameter reached 1-2 cm, the rabbit entered the experiment. This MRI protocol was repeated next day after VDA therapy.

### Digital subtraction arteriogram (DSA)

The DSA was performed with an angiography suite (Ardacis Varic, Siemens, Germany) by an interventional way 20. The right groin area of the rabbit was prepared and the longitudinal incision of skin was made above the femoral artery. The femoral artery was then obtusely separated to be completely exposed. A 5F sheath (Cook, Inc., Bloomington, Indiana) was inserted into femoral artery with Seldinger technique, then a 3F micro-catheter (Terumo, Japan) was coaxially inserted into hepatic artery guided by a 0.014-inch micro-guidewire (Transend; Boston Scientific, Miami, Florida). Hepatic arterial angiography was performed by injection of iodine contrast agent (Iopromide 300 mg/ml; Bayer Schering Pharma AG, Germany) with micro-catheter. DSA was performed both before and after transarterial delivery of VDA during a period of one hour (Fig 2b).

### Drug preparation and administration

After pre-treatment DSA, C118P powder was diluted in physiological saline to make a solution of 0.5 mg/ml. The drug was administered at a dosage of 5.0 mg/kg through micro-catheter with a syringe pump in 30 minutes. A post-treatment DSA was performed to assess whether C118P could exert an effect of tumoral vascular shutdown.

### Digital X-ray microangiography

After completion of post-therapeutic MRI, the rabbit was euthanized by iv overdose of anesthetics. Before excision of the liver, the hepatic artery was injected with barium sulfate suspension (Micropaque®, Guerbet, France). The magnified hepatic angiography was performed on the excised liver using a clinical digital mammography suite (Mammomat, Insperation, Siemens) at 28 kV and 18.0 mAs. The whole liver specimen was photographed and submerged into 10% PBS buffered Formalin for 2 weeks of fixation. Then, the tumor with surrounding liver tissue was sampled in planes similar to that of MRI and sliced into 3 mm sections, which was again radiographed and photographed for co-localization of imaging-histopathology.

### Histopathologic examination

**Figure 3:**
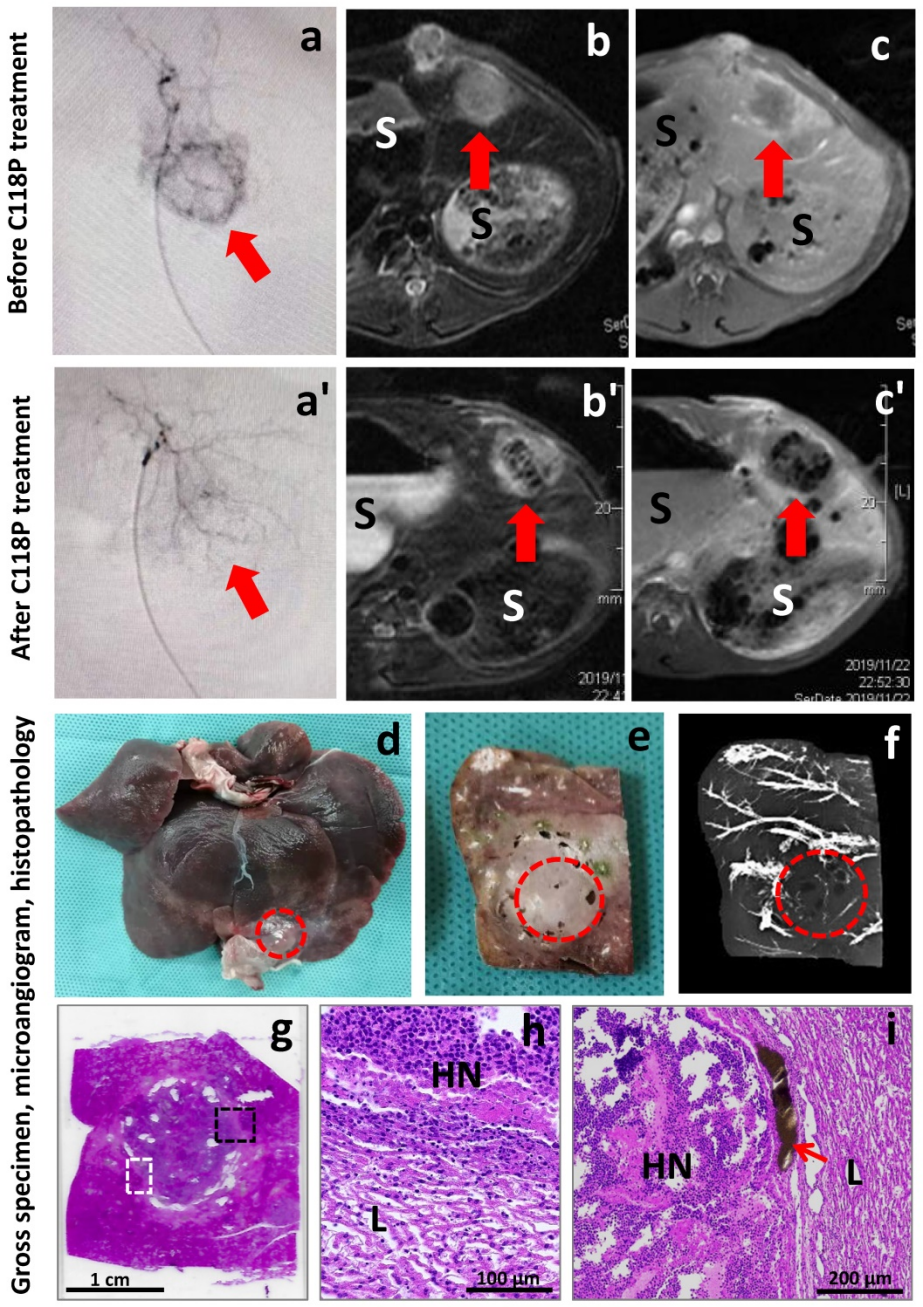
** Imaging-microangiogram-histology co-localization of a rabbit with liver implanted VX2 carcinoma treated by intrahepatic arterial infusion of a vascular disrupting agent C118P.** Before therapy, a circular tumor staining (arrow) appeared on DSA (a); the liver VX2 tumor (arrow) looked hyperintense on T2w-MRI (b); and inhomogeniously enhanced (arrow) on CE-T1w-MRI (c). After ia C118P infusion, the tumor staining (arrow) disappeared on DSA (a'); and next day the VX2 tumor (arrow) became centrally hypointense on T2w-MRI (b') and hypoenhanced on CE-T1w-MRI (c'), suggesting VDA-induced hemorrhagic intratumoral necrosis, “S” indicates stomach. At autopsy, the VX2 tumor (dashed circle) located near the edge of left lateral liver lobe (d); looked pale on fixed tissue block (e) and appeared unopaque on microangiogram (f). Histopathologically, the white and black dashed frames on H&E stained slide (g) display the focuses (h, i) of the interface between the surrounding liver (L) and VX2 tumor of nearly complete hemorrhagic necrosis (HN). The patent blood vessels filled with brownish barium particles (i, arrow) exist only at periphery of the tumor.

The 3 mm liver tissue blocks including the tumor and surrounding normal liver were processed with paraffin embedding and microtome sectioning for next hematoxylin-eosin (HE) staining, light microscopic observations and digital photography in order to correlate details with in vivo MRI, DSA, postmortem gross specimen, tissue section and their microangiography (Fig 3). Special attention was paid to interpret the histological alterations of the liver tumor after a relatively short term (24h) following C118P treatment, which on H&E staining looks different from the typical coagulation necrosis occurring a few days after interventional ablative therapies 21 as described in the literature or textbook.

## Results

All animals tolerated well this study. A typical example among currently experimented rabbits is demonstrated in Figure 3. Before treatment, the engrafted VX2 liver tumor appeared as a spherical lesion on DSA with vascular staining stronger in its periphery than that at the center (Fig 3a). On T2WI, the VX2 tumor was hyperintense relative to the surrounding liver parenchyma (Fig 3b). On CE-T1WI, tumor signal intensity could be enhanced by the injected gadolinium based contrast agent, and the tumor periphery was in a greater CE degree in comparison to the central region (Fig 3c), which resembles what was shown on DSA (Fig 3a).

Sixty minutes after intra-arterial infusion of C118P, the VX2 tumor tended to disappear from the liver on DSA with only residual vascular staining (Fig 3a'), suggesting VDA induced tumoral vascular shutdown. On MRI acquired 24h after the treatment, the VX2 tumor became a hyperintense mass but with massive intratumoral hypointense zone on T2WI (Fig 3b'), indicative of hemorrhagic necrosis, which is typically seen among solid tumors treated by VDAs. On CE-T1WI, the signal intensity of the entire tumor was virtually unenhanced in comparison to that of surrounding liver (Fig 3c'), suggesting powerful selective anticancer effect with the novel VDA C118P, which took place only overnight and by only one dosage with the liver unaffected. The intratumoral heterogeneous hypointensity on T1WI is likely due to hemosiderin released during hemorrhagic necrosis that causes T2 shortening or T2* effect.

Macroscopically, the VX2 tumor located near the edge of left lateral liver lobe almost unseen with only a few whitish spots of barium contrast agent from the surface of the liver specimen (Fig 3d). On the surface of 3 mm liver section, the tumor looked greyish without much barium stain but with sporadic cyst-like lesions (Fig 3e). On the corresponding microangiogram of this liver section, the tumor showed much less barium staining relative to the opaque vessel branches in the surrounding liver parenchyma (Fig 3f), suggesting C118P induced tumoral vascular shutdown and supporting the finding from CE-T1WI (Fig 3c'). By careful inspection, there existed a few hypodense spots surrounded by minute barium staining, which is likely related to the sporadic cyst-like lesions seen on the tissue section (Fig 3e).

Histopathologically, the C118P treated and darker stained VX2 tumor is well demarcated from the surrounding liver tissue on H&E stained slide (Fig 3g) with a few sporadic cyst-like lesions consistent to that on tissue section (Fig 3e) and microangiogram (Fig 3f). The necrotic tumor should have appeared more eosinophilic (pinker color in contrast to the more haematoxyphilic liver tissue) as conventionally seen, but due to the extremely quick tumoricidal effect with VDAs including C118P, there is simply insufficient time for the enriched oncocytic nuclei's substances including DNA to be degraded and cleared from the necrotic tumor only overnight post-therapy, a “ghost” phenomenon also true for the observations under a light microscope below.

Microscopically, by focusing on the interfaces between the tumor and adjacent liver parenchyma as marked by dashed frames on Figure 3g, typical hemorrhagic necrosis occurs in >90% of the tumoral area with inflammatory infiltration at peripheral tumor and compressed hepatocytes due to tumor expanding growth (Fig 3h,i). The VX2 tumor tissue becomes disrupted with karyopycnosis in carcinoma cells and disappearing cytoplasm. There is no patent, but only congested or thrombotic intratumoral blood vessels. The functional blood vessels are mainly found at the periphery, filled with injected brownish barium particles (Fig 3i). The suspected cystic structures are confirmed but unseen here.

## Discussion

In this study, by using a rabbit liver VX2 tumor model, the therapeutic effect of a VDA via local artery infusion was evaluated by in vivo DSA and MRI as well as ex vivo microangiography and histopathology, which allowed intraindividual comparison on pre- and post-therapeutic status of the tumor and imaging-histopathological correlation.

C118P was applied as a representative of all VDAs in this study 17. Like CA4P 1-7, the anticancer spectrum of C118P proves broad due to the fact that the target of all VDAs is the abnormal endothelium of tumoral blood vessels, which is common to all solid cancers across different species. Indeed, besides ovarian and lung cancers in patients undergoing phase I clinical trial with multiple iv dosages 17, for the first time we are able to demonstrate a dramatic tumoricidal effect in rabbits bearing implanted intrahepatic VX2 carcinoma by means of a single dose trans-catheter arterial infusion of C118P as shown in this paper. This has been realized upon a preclinical methodology for meticulous imaging-histopathology correlation, which is deemed to always yield reliable and long-lasting experimental conclusions 22.

The structure of C118 modified from CA4P was designed to avoid the potential generation of quinone metabolites in vivo, by which the efficacy and safety were supposed to be improved 17. Although normally reversible, cardiovascular toxicity proves to be a most prominent side effect among all VDAs including C118P and CA4P 1-6, which is apparently dose-dependent.

Therefore, the purpose of choosing transarterial approach for drug delivery of VDA in this study is three-fold: first, by at least half the C118P dose as opposed to its systemic dose, we expected to significantly reduce cardiovascular toxicity as well as all other potential systemic toxicity or side effects 16; secondly, we sought to clarify whether transarterial approach might impair VDAs' potency, as implied by an earlier study 15, for tumoral vascular shutdown as commonly seen with iv administration of VDAs 7-10; and lastly, this was the first step in our efforts to develop a safer dual targeting locoregional strategy of OncoCiDia 7-13.

A small dose of C118P infused through hepatic artery caused nearly complete destruction of tumoral vasculature within only 60 min as convincingly documented by DSA. The massive VX2 tumor necrosis was suggested by MRI in 24h and further confirmed by ex vivo microangiogram and histopathology. To the best of our knowledge, it is the first time that such a striking pharmacological anticancer, or tumor chemo-ablation, effect can be so vividly exhibited. Mechanistically however, it would be very much unlikely that such a mono-drug therapy will lead to cancer cure, and consequently tumor relapse would be unavoidable. But, the residual surviving cancer cells could likely be eradicated by a subsequent intravenous or transarterial dose of radioactive necrosis-avid compound such as ^131^I-Hyp that emits both high energy beta particles to destroy the DNA of cancer cells and gamma rays to facilitate nuclear scintigraphic cancer imaging 7-10.

As demonstrated by the hard imaging-histopathology correlational evidences in this typical case, we may draw the following preliminary conclusions: 1) if converted to transarterial route, the dosage of C118P could be significantly reduced without compromising its anticancer vascular disrupting property; and 2) transcatheter locoregional delivery of VDA in combinatory or mono manner could be a promising alternative to systemic VDA administration for certain clinical indications such as the treatment of solid primary and secondary malignancies in the liver, lungs, kidneys and brain, for instance.

What is the minimal effective transarterial dose of C118P? What is the minimal effective radiation dose of the iodinated necrosis-avid agent if it is also transarterially delivered? Can regional eradication be really achieved if locoregional OncoCiDia is implemented? These are the questions to be answered by our ongoing comprehensive experimental research projects.

### Funding

This work has partially been supported by the National Hi-Technology Research and Development Program (No.2012AA022701); the National Natural Science Foundation of China (No. 81771944, 81530053, 81471685, 81603142, 81771870); Liaoning Innovative Talents Supporting Program, Shenyang Innovative Talents Supporting Program (No.RC170048); Construction project of Shanghai Key Laboratory of Molecular Imaging (18DZ2260400); Shanghai Municipal Education Commission (Class II Plateau Disciplinary Construction Program of Medical Technology of SUMHS, 2018-2020); National Key Scientific and Technologic Project (Class 1.1 new anticancer drugs, No.2018ZX09301-014-006, research on C118P) at Sanhome Pharmaceutical Co. Ltd. Nanjing; and the CZC Pharmaceutical and Technology Co., Ltd. Nanjing, China.
